# Age-dependent modulations of hemispheric small-world networks in different eye states: resting-state electroencephalogram research

**DOI:** 10.1007/s11357-025-02042-5

**Published:** 2025-12-20

**Authors:** Chang Xu, Chuqiao Li, Tong Cui, Shaoqi Li, Xiaodong Han, Boye Wen, Cuibai Wei

**Affiliations:** 1 Department of Neurology, Innovation Center for Neurological Disorders, Xuanwu Hospital, Capital Medical University, Beijing, China; 2 College of Integrated Traditional Chinese and Western Medicine, Changchun University of Chinese Medicine, Changchun, China; 3 Jinan Hospital of Xuanwu Hospital, Shandong First Medical University, Jinan, China; 4 Xuan Wu Hospital of the Capital Medical University, 45 Changchun Street, Beijing, 100053 People’s Republic of China

**Keywords:** Aging, EEG, Functional connectivity, Small world, Resting state, Hemispheric asymmetry

## Abstract

Normal aging triggers substantial topological transformation within resting-state electroencephalography (rs-EEG) networks. Given that the small-world (SW) architecture of hemispheric EEG networks may reflect an evolutionarily and developmentally optimized organization, investigating their modulations with age offers critical insights into the dynamic reorganization of functional connectivity (FC) patterns throughout adulthood. Utilizing rs-EEG data from a single-site public repository collected under eyes-closed and eyes-open conditions, we employed graph-theory methods to delineate age-related alterations in the SW architecture of hemispheric functional networks across a broad adult age range (20–70 years). In 539 cognitively intact individuals, undirected weighted FC networks were constructed using eLORETA-based lagged linear connectivity for each hemisphere and three subnetworks: default mode network (DMN), frontal network (FN), and attentional network (AN). Further quantitative analyses revealed that age-related changes in SW metrics were observed exclusively in the right hemisphere, evident during eyes-closed rest and in network reconfiguration upon eye opening (ΔSW). Right-hemispheric SW and ΔSW showed frequency- and network-specific modulation with age: in the alpha2 band, all three subnetworks exhibited increasing trends, with U-shaped trajectories in DMN and AN and a linear rise in FN. In other frequency bands, specific SW metrics displayed an inverted-U curve relationship with age (DMN: alpha1 SW, delta ΔSW; FN: gamma SW; AN: theta, alpha1, gamma SW, theta ΔSW) in later adulthood, whereas beta2 SW and gamma ΔSW in FN decreased linearly. These findings revealed a right-lateralized pattern of age-related changes in intrinsic SW architecture and provided an eyes-state-dependent baseline for evaluating hemispheric network impairments in age-related neuropsychiatric disorders.

## Introduction

Health aging is generally accompanied by an evolving process of functional brain reorganization [1]. Growing evidence has suggested that age-related functional change is related to the multiscale network of complex activity patterns instead of an ensemble of an isolated brain region [2]. As a powerful technique in neurofunctional imaging, resting-state electroencephalography (rs-EEG) has been widely employed to investigate intrinsic neuronal activity in brain networks due to its sub-second time resolution, cost-effectiveness, and noninvasive nature [3]. In addition, resting-state functional magnetic resonance imaging (rs-fMRI) is another commonly used modality for investigating neural activity [4], though it has inherent limitations in temporal resolution compared to EEG.

Recent EEG and fMRI research has increasingly focused on investigating changes in functional connectivity (FC) patterns in the older brain [5–11]. Here the term “FC” in neuroscience refers to various measures of how neural activity in one brain area relates to activity in another [12, 13]. To assess whether the FC patterns reproduce the theoretically efficient and flexible networks organization, graph theoretical approaches were applied to characterize the topology of brain functional connectome [14]. In this framework, the functional network is modeled to a graph, with nodes and edges denoting the brain regions and the FC values between them, respectively [15]. Among graph theory metrics derived from resulting FC matrix, the small-world (SW) index—defined as the ratio of normalized clustering coefficient to normalized path length—is a widely adopted metric that represents the balance between local segregation (high clustering) and global integration (short path length) [16], and demonstrates high sensitivity to age-related effects [17]. Previous studies have confirmed that SW characteristics could be modified by age and neurodegeneration in a frequency-dependent manner, typically showing reduced small-worldness in both low (delta and/or theta) and high (beta and/or gamma) EEG frequency bands, and increased values in the alpha band [11, 17–20]. In other words, physiological and pathological aging systematically reshapes the topological features of oscillatory brain networks across frequency bands. Given that different oscillatory frequency bands may subserve different functional properties [21], EEG-based network analyses that reveal frequency-dependent features inaccessible to fMRI are ideally positioned to offer a refined and functionally compelling depiction of age-related brain reorganization.

Although the significance of the SW index and its modulation across canonical EEG frequency bands during aging is well established [22], research to date has largely focused on whole-brain networks or global assessments of specific cognitive networks [18–20, 23], leaving the intricate dynamics at the hemispheric and subnetwork level largely uncharted. However, the asymmetry between the two hemispheres, known as hemispheric lateralization, is considered an evolutionarily conserved mechanism of the human brain that orchestrates the dominant processing of specific cognitive tasks, enabling rapid, efficient, and specialized information processing [5, 24–26]. Investigating age-related changes in FC patterns at the hemispheric level is essential for gaining deeper insights into the neural substrates underlying aging and neurodegenerative diseases. Analyzing the rs-EEG data from 170 cognitively healthy elderly, Vecchio et al. reported that there is a more specific involvement of left-hemispheric subnetworks with intrinsic characteristics (higher SW of delta, theta, and gamma bands in the attention network (AN) and default mode network (DMN), gamma band in the frontal network (FN)) [11]. Regrettably, the narrow age range precluded a thorough characterization of how these parameters evolve across the adult lifespan. While other studies have characterized several age-related features of rs-EEG signals in older and younger adults [27], few have addressed the key “missing window” of middle adulthood (i.e., beyond just younger vs. older adults) [28], and fewer still have systematically explored the modulatory effect of age on the hemispheric SW architecture throughout adulthood. To fill this gap, the present study included a large cohort of young and middle-aged subjects to test whether any effects of age on hemispheric small-worldness are gradual across the adult age spectrum, or become exaggerated (or only appear) with advancing age.

Another critical yet underexplored dimension concerns eye-state dependence. The eyes-closed (EC) resting state is one of low EEG arousal, with the change to eyes-open (EO) primarily involving an increase in arousal [29]. The drop in alpha-band amplitude from EC to EO, as suggested by power spectrum analysis, is a principal indicator of EEG reactivity to this arousal shift, possibly reflecting the uncoupling of extensive thalamocortical interactions supporting visual processing [30]. Although reports of healthy aging impacts on the EC/EO effects remain limited, current evidence suggests that the EC-to-EO reduction in alpha power diminishes with healthy aging [27, 31]. Importantly, reduced baseline alpha EC/EO reactivity has been shown to predict poorer global cognitive and executive performance in older adults at follow-up [32]. In the context of these sparse reports, investigating EEG reactivity systematically throughout adulthood is fundamental for revealing the neural underpinnings of inter-individual differences in cognitive functioning. On the other hand, in healthy elderly controls, the SW changes between EC and EO states (∆SW) exhibited distinct frequency-dependent patterns, reflecting differential EEG network responses to visual stimulation in the classical frequency ranges. At the whole-brain level, older adults exhibited reduced SW in the EC state relative to the EO state within the alpha band, whereas SW was higher in the EC state compared to EO in the delta, beta, and gamma bands [20, 33]. Nevertheless, little is known about how such state-dependent SW alterations (i.e., network EC/EO reactivity) evolve across the broader adult age spectrum. Collectively, probing hemispheric SW characteristics across different arousal states (eyes-closed, eyes-open, post-eye-opening) may offer a more comprehensive framework for capturing age-related modulations in large-scale network topology.

Building upon previous findings, this study focused specifically on three high-level cognition-related networks—the default mode network (DMN), frontal network (FN), and attentional network (AN)—which exhibit hemispheric-specific SW architecture across different EEG frequency bands [11]. We employed rs-EEG combined with graph-theoretical analyses to systematically investigate age-related modulation of small-worldness in these three subnetworks, introducing three key innovations: (1) probing hemispheric rather than relatively generalized whole-brain networks, (2) comprehensively evaluating age-dependent effects across eyes-closed, eyes-open, and post-eye-opening conditions, and (3) spanning a broad adult age range (20–70 years) to incorporate the previously underrepresented middle adulthood. Leveraging a large-scale population cohort, this study advances a more nuanced understanding of brain aging and facilitates the establishment of eyes-state-dependent normative datasets to support future clinical assessment of hemispheric network alterations in patients with clinically relevant disorders.

## Materials and methods

### Participants

We included 608 cognitively intact participants (age: mean = 44.1, SD = 14.5, range 20–70 years; sex: F = 376, M = 232) from the Dortmund Vital Study [34, 35] in this study. The Dortmund Vital Study was conducted by the Leibniz Research Centre for Working Environment and Human Factors at the Technical University Dortmund (IfADo) (ClinicalTrials.gov Identifier: NCT05155397), approved by the local ethical committees in accordance with the declaration of Helsinki. All participants have given signed informed consent for their participation. The study population self-reported as healthy was recruited from local companies and public institutions and through advertisements in newspapers and public media. Exclusion criteria were a history of severe mental or neurological disorders, other systemic diseases (such as cardiovascular, oncological, and eye diseases), severe head injuries, and the use of neuroleptics or psychotropic drugs. Participants whose preprocessed EEG recordings failed to meet the required signal quality standards were excluded from this study. Ultimately, data from 539 subjects (age: mean = 43.7, SD = 14.6, range 20–70 years; sex: F = 332, M = 207) were entered into the final analysis. Raw EEG data and accompanying demographic details (including age, gender, and handedness) can be accessed from https://openneuro.org/datasets/ds005385/versions/1.0.2.

To examine age-related effects, participants were stratified into five consecutive 10-year age groups spanning 20 to 70 years (20–29, 30–39, 40–49, 50–59, and 60–70), with a reasonably balanced distribution across groups (*χ*^2^(4) = 6.71, *p* = 0.15). In addition, gender ratios did not differ significantly among the five age groups (χ^2^(4) = 9.04, *p* = 0.06) (Table 1).

**Table 1:** Demographics. Summary of each age group, including sample size (*N*), mean age ± s.d., and sex distribution (F/M)

Age group	*N*	Age (mean ± s.d.)	Sex (F/M)
20–29	**125**	**25.07(2.74)**	**88/37**
30–39	**103**	**33.52(2.78)**	**55/48**
40–49	**96**	**44.94(3.21)**	**63/33**
50–59	**119**	**54.23(2.99)**	**76/43**
60–70	**96**	**64.52(3.31)**	**54/42**

### Data recordings and preprocessing

For each participant, rs-EEG data was recorded using a 64-channel Brain Amp DC amplifier (Brain Products, Germany) for three minutes under both eyes-open and eyes-closed conditions, administered before and after a 2-h session of cognitively demanding tasks. Electrodes were positioned according to the extended 10–20 system [36]. Signals were online filtered with a 250 Hz DC cutoff and digitized at a sampling rate of 1000 Hz. A common mode sense (CMS) active electrode and a driven right leg (DRL) passive electrode were employed to form a feedback loop, thereby stabilizing the average potential. Reference and ground electrodes were integrated within the CMS-DRL loop. To minimize potential task-related influences, only eyes-open and eyes-closed data acquired prior to the cognitive tasks were included in the subsequent analyses.

EEG preprocessing was performed in MATLAB R2018b (MathWorks, Natick, MA) using custom scripts based on the EEGLAB 2019.1 toolbox (Swartz Center for Computational Neuroscience, La Jolla, CA; http://www.sccn.ucsd.edu/eeglab). To ensure comparability with previous EEG connectivity studies [11], a subset of electrode channels corresponding to the standard 19-channel montage (Fp1, Fp2, F7, F8, F3, F4, T3, T4, C3, C4, T5, T6, P3, P4, O1, O2, Fz, Cz, and Pz) was selected, a configuration also widely adopted in clinical practice [37–40]. Offline preprocessing included band-pass filtering (1–45 Hz) and notch filtering (48–52 Hz) to remove artifacts and line noise. The continuous EEG was segmented into 2-s epochs. Datasets with one or more bad channels were excluded from further analysis (final N = 63). Remaining data were visually inspected to identify and remove contaminated segments. Independent component analysis (ICA) was then conducted using the Infomax (runica) algorithm [41], which enables the separation and removal of artifacts such as eye movements, cardiac activity, and scalp muscle contraction [42, 43]. Artifact-corrected EEG was finally re-referenced to the common average for subsequent network analyses.

### Functional connectivity of cortical source analysis

EEG connectivity analysis has been performed using the exact low resolution electromagnetic tomography eLORETA [44] http://www.uzh.ch/keyinst/NewLORETA/LORETA01.htm.The eLORETA algorithm is a single linear inverse solution for EEG signals that has the property of exact localization to test point sources under ideal (noise-free) conditions [45]. Interregional connectivity values were estimated using the Lagged Linear Coherence (LagR) algorithm implemented in eLORETA, based on regions of interest (ROIs) defined according to the Brodmann areas (BAs) [46].

**Fig. 1:**
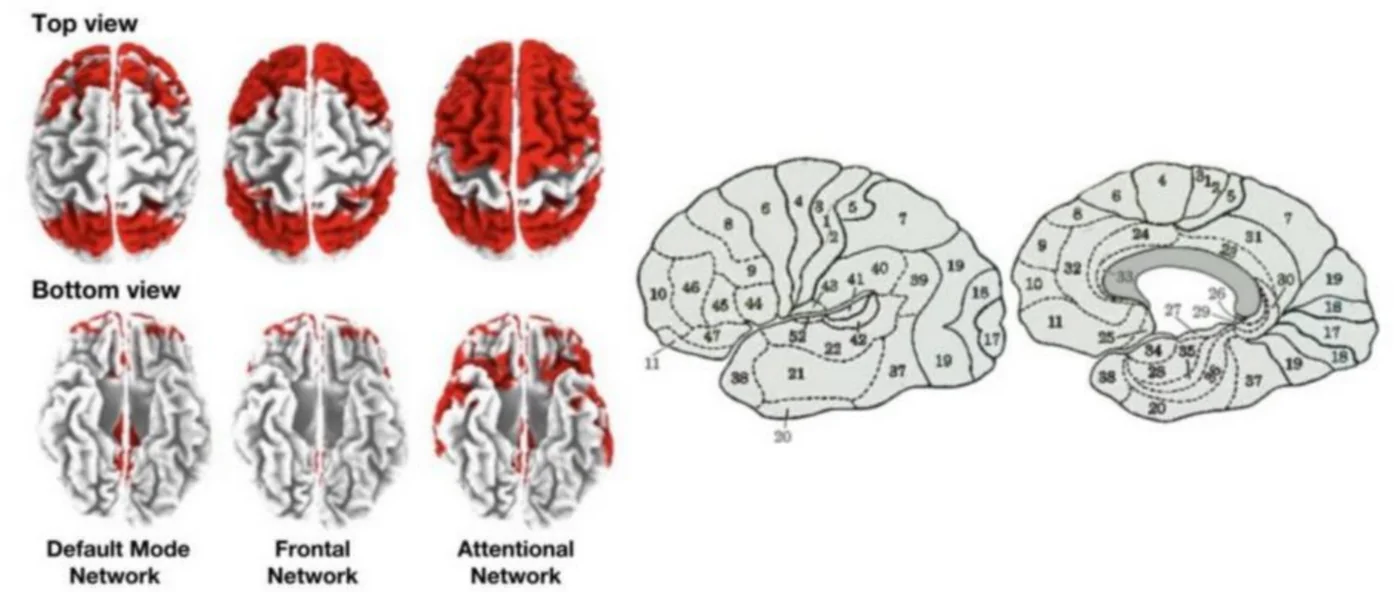
Schematical illustration of considered networks. Default Mode (DMN)–Brodmann Areas (BA) 7, 8, 10, 23, 31, 32, 39, 46; Frontal (FN) –BA2, 7, 8, 9, 10, 40, 44, 45, 46; Attentional (AN) –BA6, 7, 8, 9, 10, 21, 22, 31, 32, 39, 40, 45, 47

The built-in Brodmann template divided each hemisphere into 42 ROIs (BAs 1, 2, 3, 4, 5, 6, 7, 8, 9, 10, 11, 13, 17, 18, 19, 20, 21, 22, 23, 24, 25, 27, 28, 29, 30, 31, 32, 33, 34, 35, 36, 37, 38, 39, 40, 41, 42, 43, 44, 45, 46, 47). ROIs included in the three specific cognitive subnetworks examined in the present study are as follows [11, 47]: 8 ROIs in the DMN (BAs 7, 8, 10, 23, 31, 32, 39, 46); 9 ROIs in the FN (BAs 2, 7, 8, 9, 10, 40, 44, 45, 46); 13 ROIs in the AN (BAs 6, 7, 8, 9, 10, 21, 22, 31, 32, 39, 40, 45, 47). Each resting-state subnetwork was delineated separately in the left and right hemispheres (see Fig. 1).

For each subject, the mean eLORETA current density time series was computed by averaging all voxels within a 19-mm radius sphere centered at each BA of interest. LagR was subsequently computed for all possible ROI pairs within each resting-state hemispheric subnetwork across canonical EEG frequency bands [48]: delta (2–4 Hz), theta (4–8 Hz), alpha1 (8–10.5 Hz), alpha2 (10.5–13 Hz), beta1 (13–20 Hz), beta2 (20–30 Hz), and gamma (30–45 Hz). Starting with the definition of the complex valued coherence between time series x and y in the frequency band w, which is based on the cross-spectrum given by the covariance and variances of the signals, the equation that calculates the LagR in the frequency band *w* is as follows [44]:

$${\mathrm{LagR}}_{\mathrm{xyw}}^{2}=\frac{{\left[\text{Im Cov }\left(\mathrm{x},\mathrm{y}\right)\right]}^{2}}{\text{Var }\left(\mathrm{x}\right)\cdot \mathrm{Var}\left(\mathrm{y}\right)-{\left[\text{Re Cov }\left(\mathrm{x},\mathrm{y}\right)\right]}^{2}}$$

(x and y: time series of two BAs, Im and Re: imaginary and real parts, Var and Cov: variances and covariance).

The connectivity matrices are composed of n x n elements, where n is the number of nodes (BAs within each network) and the n_ij_ element is the connectivity value between the i-th and the j-th node. The LagR measure, which represents the connectivity value, minimizes spurious zero-lag instantaneous interactions caused by volume conduction and thereby enhances the physiological validity of EEG-based FC estimates [46]. The LagR values computed between all pairs of ROIs within each network, for each frequency band and each subject, were used as the edge weights in the subsequent graph analyses.

### Graph analysis

In graph theory, networks are constituted by a set of nodes (vertices) and links (edges) between pairs of nodes, allowing a mathematical representation of a real-world complex system [49]. In this study, we constructed the three undirected and weighted subnetworks (DMN, FN, and AN) within each hemisphere, where nodes were defined as the aforementioned BAs and edges were weighted by the LagR values calculated between nodes within each network [50]. Once built, networks' two core measures of graph theory were computed: weighted clustering coefficient (*C*^w^) and weighted characteristic path length (*L*^w^), representative of local interconnectedness and global connectedness, respectively [51].

The *C*^w^ of a node is defined as the normalized sum of geometrically averaged edge weights of all triangles associated with the node [13]. The corresponding calculation formula is as follows:

$${C}^{w}=\frac{1}{n}\sum_{i\in N}\frac{2{t}_{i}^{w}}{{k}_{i}\left({k}_{i}-1\right)}$$

where ${k}_{i}$ is the degree of node i, ${t}_{i}^{w}$ is the geometric mean of triangles surrounding node *i*, ${t}_{i}^{w}=\frac{1}{2}\sum_{j,h\in N}{\left({W}_{ij}{W}_{ih}{W}_{jh}\right)}^{1{~}^{1}\!\left/ \!{~}_{3}\right.}$. ${W}_{ij}$ are connection weights associated with links (*i*, *j*), assuming that weights are normalized, such that 0 ≤ ${W}_{ij}$ ≤ 1 for all *i* and *j*. 

L^w^ is defined as ${L}^{w}=\frac{1}{n}\sum_{i\in N}\frac{\sum_{j\in N,j\ne i}{d}_{ij}^{w}}{n-1}$ with ${d}_{ij}^{w}=\sum_{{a}_{uu}\in {g}_{i}^{w}\to j}f\left({w}_{uu}\right)$ that represents the shortest weighted path length ${g}_{i\leftrightarrow j}^{w}$ between i and j [13]. is a f map (e.g., an inverse) from weight to length and is the shortest weighted path between i and j. 

At the network level, the clustering coefficient and shortest path length are defined as the mean of* C*^*w*^ and *L*^w^ over all nodes in the network. To obtain individual-level normalized network metrics, we divided the *C*^w^ and *L*^w^ at each frequency band by their respective mean values averaged across all frequency bands [11, 16, 40, 52]. The SW coefficient of each hemispheric subnetwork was then calculated as the ratio of the normalized *C*^w^ to the normalized *L*^w^. The SW index describes the balance between local connectedness and global integration within a network, with a SW > 1 indicative of small world topology (typical of real-world networks). Small-world network organization combines the high mean clustering coefficient typical of regular lattice networks (which ensure integrity within local networks that may hold specific functions) and the short path length typical of random networks (which guarantees efficient large-scale integration of subnetworks). This means that nodes are linked through relatively few intermediate steps, and most nodes are not direct neighbors.

In particular, we computed *C*^w^, *L*^w^, and SW parameters in each of the hemispheric networks described above across seven independent EEG frequency bands, adopting the estimates presented at http://www.brain-connectivity-toolbox.net, adapted by our own Matlab scripts.

### Statistical evaluation

First aim of this study was to determine whether SW properties of EEG networks exhibit hemispheric-specific patterns across age groups in different eye states. To achieve this, for each experimental condition, we conducted a three-way repeated measures Analysis of Variance (ANOVA). Specifically, SW values in the EC and EO conditions, as well as the relative difference between them (∆SW = [EC − EO]/[EO + EC]), were analyzed separately as dependent variables, with AgeGroup (20–29, 30–39,40–49, 50–59, 60–70) as a between-subjects factor, and FrequencyBand (delta, theta, alpha1, alpha2, beta1, beta2, and gamma) and Hemisphere (left, right) as within-subject factors. This global analysis allowed for the examination of overall age-related and hemispheric patterns of SW architecture across the entire brain. Second, to investigate age-related effects at a finer scale, we evaluated SW values of each hemispheric network (right/left DMN, FN, and AN) using two-way ANOVAs with AgeGroup and FrequencyBand as factors, stratified by experimental condition. For each hemisphere, we treated DMN, FN, and AN separately since these networks differed in the number of edges. This stepwise approach—from whole-brain three-factor ANOVAs to subnetwork-specific two-factor ANOVAs—provided a nuanced characterization of age-related alterations in SW organization. Finally, over the entire age range studied, generalized additive models (GAMs) with penalized splines were applied to determine whether the significant age-related SW changes in hemispheric subnetworks identified by ANOVAs followed linear or nonlinear trajectories.

To evaluate whether the contributions of C to age-related differences in SW were comparable to, or significantly distinct from, those of L, one-way ANOVAs were conducted separately for C and L, focusing exclusively on SW values that exhibited significant age-related effects under a given eye state and within specific frequency bands of the corresponding hemispheric networks.

The compliance of dependent variables with ANOVA’ s assumptions (normality, homogeneity of variance) was not always met. Nevertheless, the test is sufficiently robust against departure from these assumptions to allow for its use [53]. Greenhouse and Geisser correction was employed to adjust for potential violations of the sphericity assumption in the repeated measures ANOVA when needed (Mauchly’s test with *p* < 0.05) [54]. Besides, all significant effects in ANOVA were followed up with Duncan test’s multiple comparisons post hoc, and the level of significance was set to *p* < 0.05. Pearson’s chi-square test was applied to examine gender ratio differences across age categories, while a chi-square goodness-of-fit test was used to assess the distribution of participants among the five age groups. All statistical analysis was performed with R statistical software (version 4.4.0).

## Results

### Comparison of age groups in left and right hemispheres

#### EC condition

**Fig. 2:**
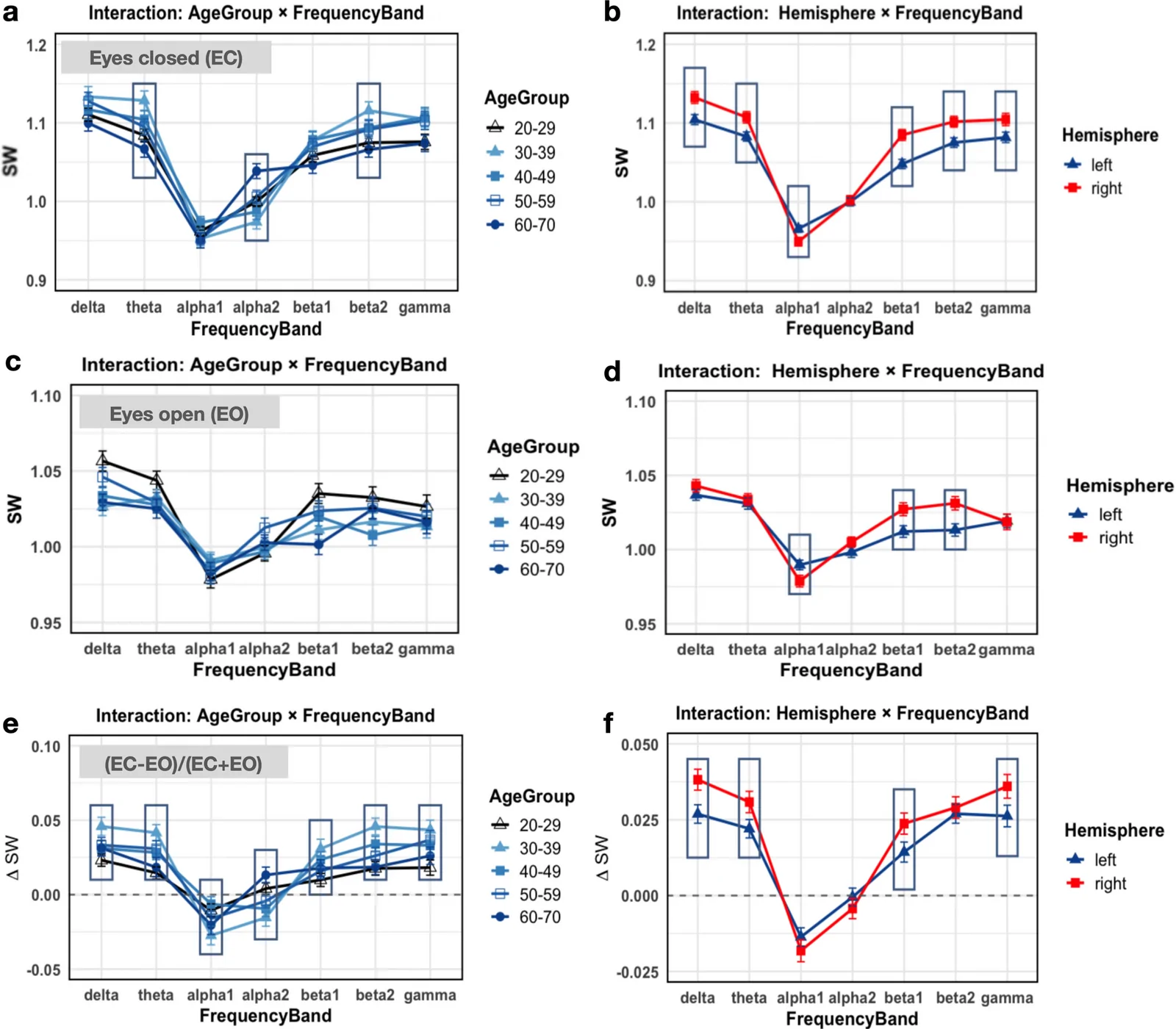
AgeGroup × FrequencyBand and Hemisphere × FrequencyBand ANOVA results for the small-world (SW) parameter of hemispheric networks in different eyes states. Left panel: AgeGroup × FrequencyBand ANOVAs for SW in the eyes-closed (EC; **a**) and eyes-open (EO; **c**) conditions, and for ΔSW during reactivity to eye opening (**e**). Right panel: Hemisphere × FrequencyBand ANOVAs for SW in EC (**b**) and EO (**d**) conditions, and for ΔSW (**f**). Frequency bands showing significant post hoc differences between age groups or hemispheres are indicated with rectangular boxes (*p* < 0.05)

In the EC condition, the 5 (AgeGroup: 20–29, 30–39, 40–49, 50–59, and 60–70) × 2 (Hemisphere: left, right) × 7 (FrequencyBand) ANOVA revealed no significant three-way interaction. Among the two-way interactions involving AgeGroup, only the AgeGroup × FrequencyBand effect reached significance (*F*(24, 3204) = 2.25, *p* = 0.006). Post hoc analysis indicated that older adults (60–70 years) exhibited higher mean SW values in the alpha2 band relative to all other age groups (20–29, *p* = 0.010; 30–39, *p* < 0.001; 40–49, *p* = 0.001; 50–59, *p* = 0.031), irrespective of hemisphere. In addition, the 50–59 group showed significantly higher alpha2-band SW than the 30–39 group (*p* = 0.034). On the other hand, in the theta and beta2 bands, participants aged 30–39 exhibited significantly higher SW coefficients relative to the 20–29 group (theta: *p* = 0.014; beta2: *p* = 0.022) and the 60–70 group (theta: *p* = 0.001; beta2: *p* = 0.009) (see Fig. 2a). In frequency bands with significant SW differences, one-way ANOVA revealed that C and L did not differ between age groups.

A robust Hemisphere × FrequencyBand interaction (*F*(6, 3204) = 10.69, *p* < 0.001) showed that, across all the age groups, SW coefficients were consistently lower in the alpha1 band and higher in the delta, theta, beta1, beta2, and gamma bands in the right hemisphere compared with the left (all *p* < 0.001; see Fig. 2b). Post hoc tests confirmed that the right hemisphere exhibited higher C and L values in the alpha1, beta1, beta2, and gamma bands, whereas no significant hemispheric differences in C and L were observed in the delta and theta bands.

#### EO condition

In the EO condition, neither three-way interaction nor AgeGroup × FrequencyBand interaction (*F*(24, 3204) = 1.32, *p* = 0.166; see Fig. 2c) reached significance. In contrast, the significant Hemisphere × FrequencyBand interaction (*F*(6, 3204) = 4.59, *p* < 0.001) revealed a frequency-dependent hemispheric asymmetry resembling that observed in the EC condition, albeit confined to a narrower range of frequency bands. This asymmetry was marked by a selective decrease of SW in the alpha1 band and increases in the beta1 and beta2 bands in the right hemisphere compared with the left (alpha1: *p* = 0.010; beta1: *p* = 0.002; beta2: *p* < 0.001) (see Fig. 2d). ANOVA on C and L in the frequency bands with significant SW differences revealed no hemispheric differences.

#### ΔSW (reactivity to eye opening)

ANOVA using ΔSW as the dependent variable—an index of rs-EEG network reactivity to eye opening—demonstrated significant AgeGroup × FrequencyBand (*F*(24, 3204) = 2.43, *p* = 0.002) and Hemisphere × FrequencyBand (*F*(6, 3204) = 3.50, *p* = 0.005) interactions, highlighting both age-related and hemispheric modulation of network dynamics. Echoing prior findings that the eyes opening elevates alpha2 SW while suppressing SW in other frequency bands, we interpret the more negative ΔSW in alpha2 alongside largerΔSW in other bands as reflecting heightened network reactivity to eye opening. As illustrated in Fig. 2e, the 30–39 years cohort exhibited maximal SW reactivity, with absolute ΔSW values consistently trending above those of other age groups across the frequency spectrum. Notably, this reactivity was significantly higher across all seven frequency bands than in the 20–29 age group (all *p* < 0.04). Moreover, in both the low-frequency theta band and the high-frequency beta2 band, participants aged 30–39 showed significantly greater SW reactivity than older groups: reactivity exceeded that of the 60–70 group in the theta band (*p* = 0.015) and was higher than that of both the 50–59 (*p* = 0.027) and 60–70 (*p* = 0.004) groups in the beta2 band. In the alpha2 band, the 60–70 age group exhibited a pronounced reversal of the SW reactivity (ΔSW > 0) relative to the other groups (ΔSW < 0) and showed significantly lower reactivity than the younger 30–39 (*p* = 0.001), 40–49 (*p* = 0.012), and 50–59 (*p* = 0.043) groups.

Inspection of meanΔSW values further indicated that the right hemisphere exhibited greater SW reactivity to eye opening across all frequency bands, regardless of age group (see Fig. 2f). This trend was corroborated by the significant Hemisphere × FrequencyBand interaction, which revealed robust right-hemisphere dominance in ΔSW across adulthood, most notably in the delta (*p* < 0.001), theta (*p* = 0.011), beta1 (*p* = 0.007), and gamma (*p* = 0.019) bands. Another noteworthy observation is that only in the alpha1 and alpha2 bands did SW values increase after eye opening (ΔSW < 0), whereas an increase (ΔSW > 0) was observed in other lower-frequency (delta and theta) and higher-frequency (beta1, beta2, and gamma) bands.

### Age-dependent modulation of SW metrics in left and right DMN

**Fig. 3:**
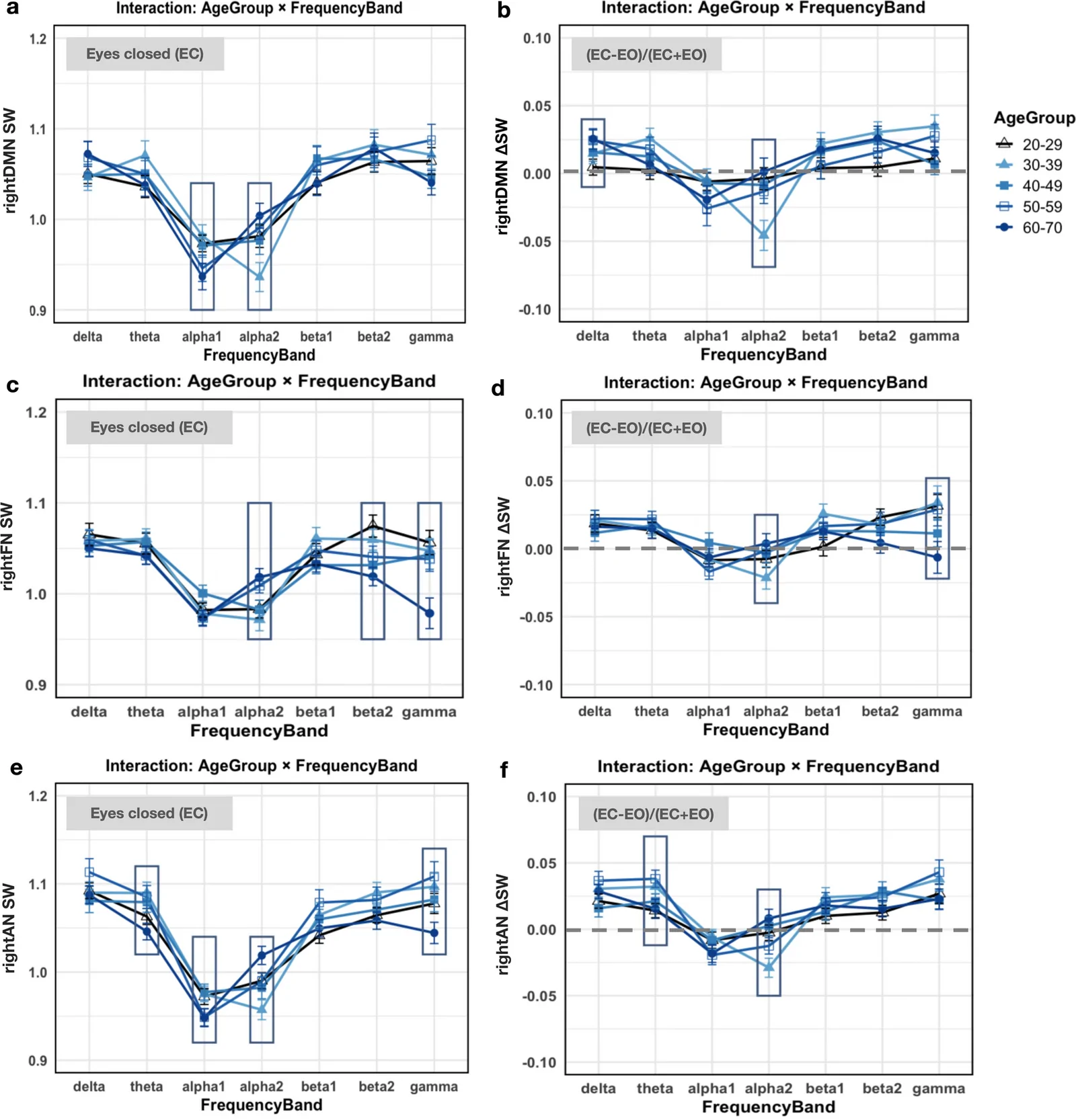
Significant AgeGroup × FrequencyBand ANOVA results for the small-world (SW) parameter of hemispheric subnetworks across different resting state conditions. Left panel: SW of right DMN (**a**), right FN (**c**), and right AN (**e**) in the eyes-closed (EC) condition. Right panel: ∆SW of right DMN (b), right FN (**d**), and right AN (**f**) during reactivity to eye opening. Frequency bands showing significant post hoc differences between age groups or hemispheres are highlighted with rectangular boxes (*p* < 0.05). AN: attentional network; DMN: default mode network; EO: eyes-open; FN: frontal network

For the DMN, neither SW values in the EC and EO conditions nor ΔSW showed significant main or interaction effects of AgeGroup in the left hemisphere. In the right hemisphere, however, SW values in the EC condition exhibited a significant AgeGroup × FrequencyBand interaction (*F*(24, 3204) = 1.68, *p* = 0.047). Post hoc Duncan tests indicated that SW values in the alpha1 band were significantly lower in the 60–70 group compared to the 20–29 (*p* = 0.032) and 30–39 (*p* = 0.012) groups. In the alpha2 band, the 30–39 group showed lower SW values than the 20–29, 50–59, and 60–70 groups (all *p* < 0.021) (see Fig. 3a). ΔSW in the right DMN also displayed a significant AgeGroup × Frequency Band interaction (*F*(24, 3204) = 1.91, *p* = 0.014). Post hoc analyses revealed that in the delta band, the 20–29 group exhibited lower reactivity (i.e., lowerΔSW) than participants aged 50–59 (*p* = 0.036) and 60–70 (*p* = 0.040). Additionally, the 30–39 group showed higher reactivity (i.e., lowerΔSW) in the alpha2 band than all other age groups (all *p* < 0.010) (see Fig. 3b). No significant AgeGroup × FrequencyBand interaction was observed for SW in the EO condition in the right DMN (*p* = 0.274).

**Fig. 4:**
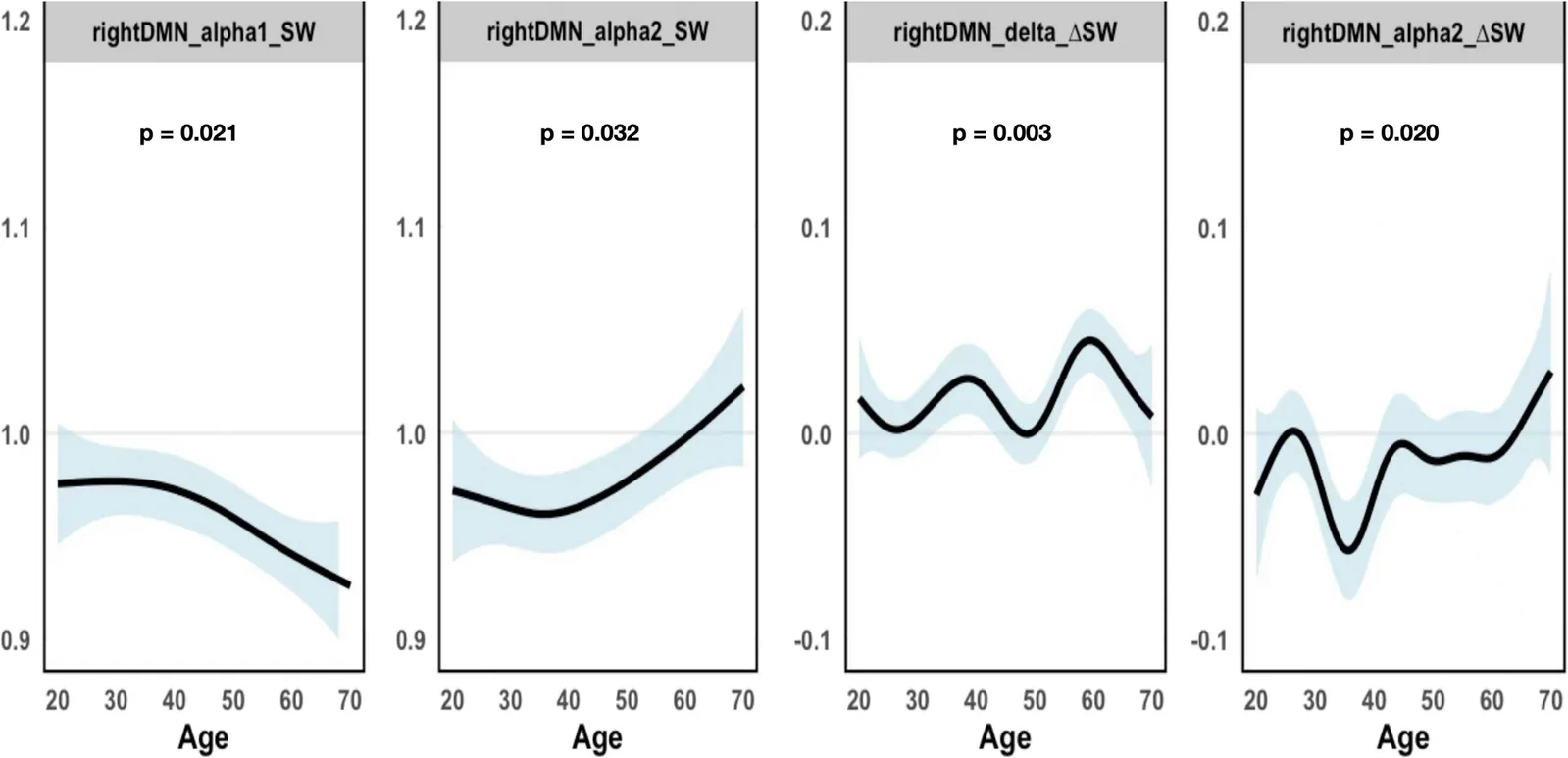
Age-related changes in the small-world (SW) parameter and ΔSW (reactivity to eye opening) of the right default mode network (DMN) in specific frequency bands, modeled with generalized additive models (GAMs). The black line represents the fitted smooth curve between variables; the light blue ribbon represents the corresponding 95% confidence interval

Given that age-related changes in SW and ΔSW do not follow a simple monotonic trend across age groups (20–29 through 60–70), GAMs were employed to further probe potential non-linear associations between age and EEG network measures in which age effects were observed. GAM analysis underscored that age-related alterations in the topological organization of the right DMN followed a non-linear trajectory (see Fig. 4). Specifically, in eyes-closed brain states, SW values in the alpha1 band remained relatively flat throughout the twenties and thirties, after which they exhibited a gradual age-related decline. By contrast, those in the alpha2 band exhibited a U-shaped trajectory, decreasing from the twenties through the thirties and subsequently rising from the forties onward. With respect to network reactivity, the delta-band ∆SW showed a wave-like course characterized by elevated levels in the sixties, while the alpha2-band ∆SW displayed a pronounced dip in the thirties followed by a gradual recovery in subsequent decades. Taken together, analyses based on both the EC condition and ΔSW offer complementary perspectives, enabling the identification of frequency-specific network patterns that characterize distinct age groups.

### Age-dependent modulation of SW metrics in left and right FN

ANOVAs revealed no significant main or interaction effects of AgeGroup on SW values in EC or EO conditions or onΔSW within the left FN. In sharp contrast, the right FN demonstrated significant AgeGroup × FrequencyBand interactions, observed for SW values in the EC condition (*F*(24, 3204) = 2.54, *p* < 0.001) as well as forΔSW (*F*(24, 3204) = 1.62, *p* = 0.045). In the EC condition, post hoc comparisons illustrated that in the alpha2 band, participants aged 60–70 exhibited significantly higher SW coefficients than those aged 20–29 (*p* = 0.014), 30–39 (*p* = 0.002), and 40–49 (*p* = 0.018), while those aged 50–59 had higher values than the 30–39 group (*p* = 0.008) (see Fig. 3c). In the high-frequency beta2 band, younger adults (20–29 years) exhibited significantly higher SW coefficients than those aged 40–70. Furthermore, individuals aged 60–70 demonstrated markedly lower SW coefficients across both the beta2 and gamma bands compared to younger groups: specifically, lower than those aged 20–39 in the beta2 band (all *p* < 0.024) and lower than those aged 20–59 in the gamma band (all *p* < 0.005). In terms ofΔSW in the right FN, the significant AgeGroup × Frequency Band interaction revealed that the 30–39 group exhibited greater reactivity (i.e., lower ΔSW) relative to participants aged 50–59 (*p* = 0.036) and 60–70 (*p* = 0.014) in the alpha2 band. In the gamma band, the 60–70 group showed poorer reactivity (i.e., lower ΔSW) than all other age groups (all *p* < 0.025) (see Fig. 3d).

**Fig. 5:**
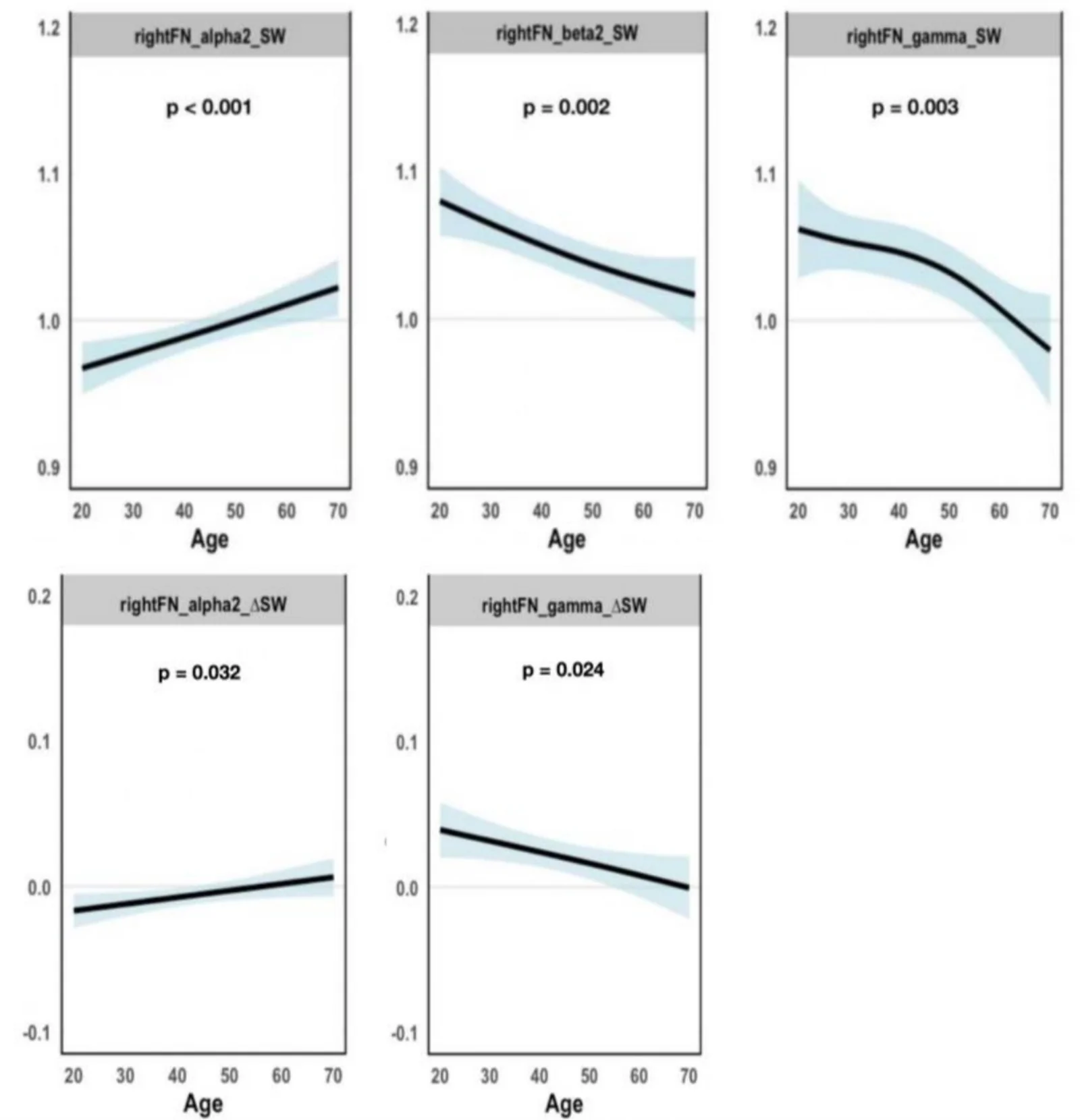
Age-related changes in the small-world (SW) parameter and ΔSW (reactivity to eye opening) of the right frontal network (FN) in specific frequency bands, modeled with generalized additive models (GAMs). The black line represents the fitted curve between variables; the light blue ribbon represents the corresponding 95% confidence interval

For SW features exhibiting significant inter-group differences, GAMs were applied to capture linear or nonlinear age effects in SW and ΔSW. Notably, in the alpha2 band, both SW values and the changes in SW upon eye opening in the right FN exhibited a linear increase with age, whereas in other frequency bands, SW or ΔSW demonstrated an opposite trend (see Fig. 5). In the EC condition, SW metrics in the beta2 and gamma bands presented distinct age-related declining trajectories: gamma-band SW values declined gradually during adulthood until the early forties and more rapidly thereafter, whereas beta2-band SW exhibited a near-linear decline throughout the entire adult lifespan. A similar trend was observed in the theta band, although this did not reach statistical significance. On the other hand, ΔSW in the gamma band, reflecting the reactivity of high-frequency EEG subnetworks to eye opening, declined linearly with age.

### Age-dependent modulation of SW metrics in left and right AN

**Fig. 6:**
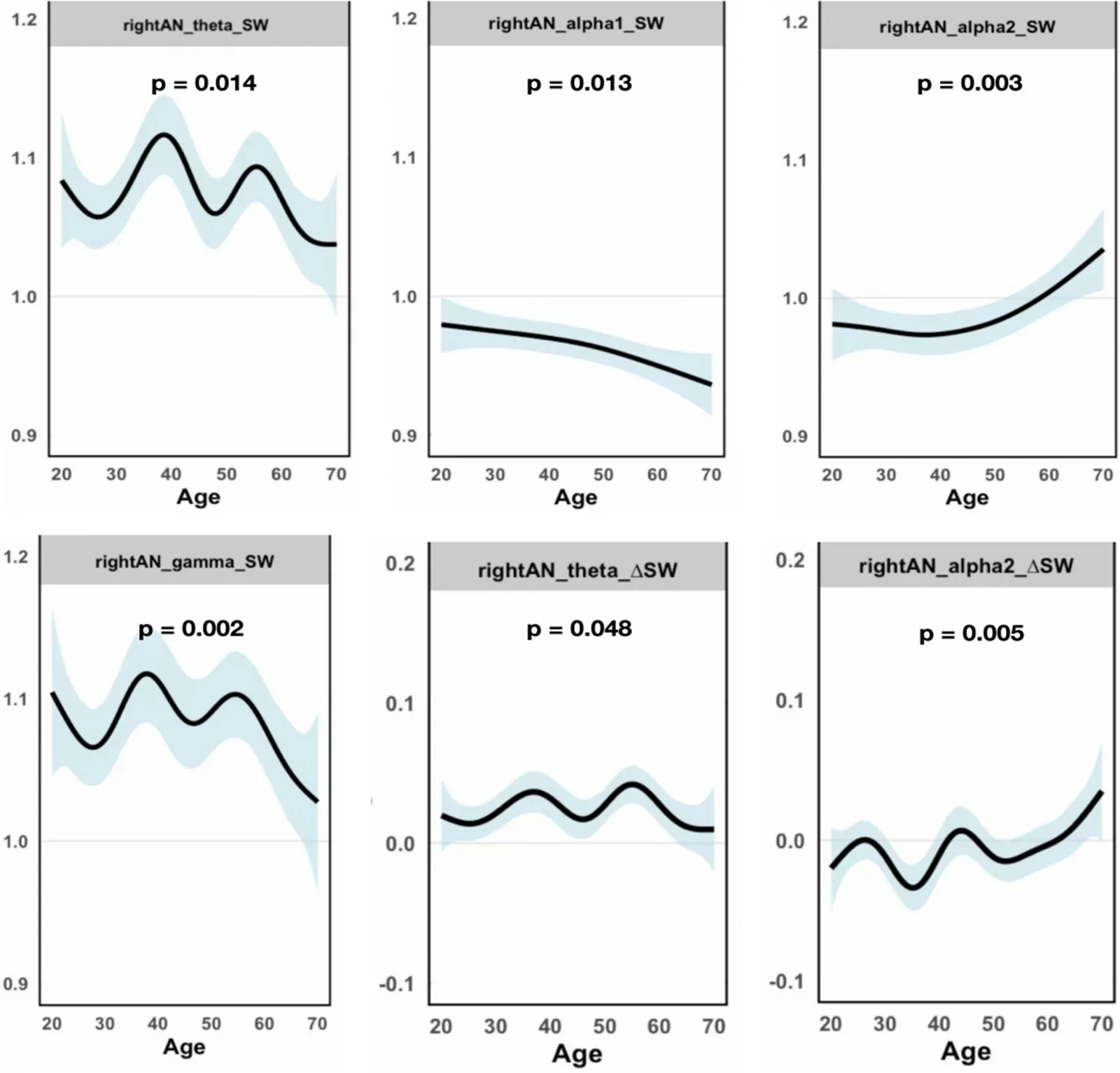
Age-related changes in the small-world (SW) parameter and ΔSW (reactivity to eye opening) of the right attentional network (AN) in specific frequency bands, modeled with generalized additive models (GAMs). The black line represents the fitted curve between variables; the light blue ribbon represents the corresponding 95% confidence interval

In the AN, age-related alterations in SW values and ΔSW were observed only in the right hemisphere across resting-state conditions, with no comparable effects in the left hemisphere. Specifically, significant AgeGroup × FrequencyBand interactions emerged for SW values in the EC brain state (*F*(24, 3204) = 2.44, *p* = 0.001) and forΔSW (*F*(24, 3204) = 2.68, *p* < 0.001). Duncan’s post hoc analysis showed lower SW values in the theta band and viceversa higher in the alpha2 band in the 60–70 years group compared to those aged 30–59 (all *p* < 0.048) (see Fig. 3b). In the alpha2 bands, SW values were elevated in the 20–29 (*p* = 0.008) and 60–70 (*p* = 0.014) groups relative to the 30–39 group, consistent with the U-shaped trajectory revealed by subsequent GAM analysis (Fig. 6). Significant and relatively consistent age effects were observed in the alpha1 and gamma bands, with younger groups exhibiting higher small-worldness compared to older groups: alpha1-band SW was higher in the 40–49 group than in the 50–70 group (both *p* < 0.039), and gamma-band SW in the 30–39 (*p* = 0.012) and 50–59 (*p* = 0.001) groups exceeded that of the 60–70 group. ForΔSW, the 50–59 group had greater reactivity (i.e., higher ΔSW) than participants aged 20–29 (*p* = 0.029), 40–49 (*p* = 0.048), and 60–70 (*p* = 0.009) in the theta band. In the alpha2 band, the 30–39 group showed the greatest reactivity (i.e., lowest ΔSW) among all age groups (all *p* < 0.029) (see Fig. 3d).

GAM analyses, consistent with observations in other rs-EEG subnetworks reported above, showed that SW and ΔSW in the right AN rose with advancing age in the alpha2 band, in contrast to a downward trend in the remaining frequency bands. In the EC condition, SW exhibited an inverted U-shaped relationship in the alpha1 band, and a U-shaped relationship with age in the alpha2 band, with inflection points at approximately 30 and 40 years, respectively. In addition, SW in theta and gamma bands followed a bimodal trajectory, with a primary peak in the thirties and a secondary rebound in the fifties, alongside an overall declining trend across adulthood. After age 50, ΔSW in the theta and alpha2 bands followed U-shaped and inverted U-shaped patterns, respectively, reflecting a decline in SW reactivity with age during middle and older adulthood.

## Discussion

This study provides the first comprehensive examination of age-related changes in SW organization of hemispheric EEG networks across adulthood, incorporating eyes-closed, eyes-open, and post-eye-opening network reconfiguration. We observed a consistent rightward predominance in SW topology across all age groups and recording conditions. From 20 to 70 years, both whole-brain and right-hemispheric subnetworks underwent marked topological reorganization, with SW in the eyes-closed state and ΔSW upon eyes opening exhibiting frequency-dependent, age-related modulations. Generalized additive model analyses further revealed that SW metrics showing significant age-group differences followed linear or nonlinear age trajectories in a frequency-specific manner.

### Right-hemispheric dominance in EEG network small-worldness

Throughout adulthood, both the right and left hemispheric EEG networks possessed SW attributes. Across all age groups and recording conditions, global analyses consistently revealed the significant Hemisphere × FrequencyBand interaction, indicating a robust rightward asymmetry in EEG network organization spanning multiple frequency bands. In the EC state, the right hemisphere showed significantly stronger SW topological properties (clustering coefficient, characteristic path length, and small-worldness) relative to the left in the high-frequency (beta1, beta2, and gamma) bands, suggesting that the right hemisphere may be more efficient in information processing [55]. By contrast, the elevation of left-hemispheric SW in the alpha1 band stemmed from reductions in both C and L, particularly in path length. The notable declines in local clustering and path length may, respectively, reflect disrupted local connectivity and increased network randomization [56], both potentially contributing to the diminished network efficiency of the left hemisphere in the alpha1 frequency band. Frequency-dependent hemispheric asymmetry in the EO state closely paralleled that observed in the EC condition, with the right hemisphere exhibiting selective increases in SW in the beta1 and beta2 bands and a decrease in the alpha1 band. Overall, a pronounced right-hemispheric dominance was observed in EEG frequency bands showing significant hemispheric differences in SW, consistent across both EC and EO conditions. Together with previous work on brain asymmetry across different age groups, rightward asymmetry in structural and functional network efficiency has been repeatedly proven in healthy adults [57–61].

On the other hand, the SW changes from EC to EO observed in our study align well with frequency-specific network dynamics and underlying neurophysiological mechanisms. The EC-to-EO increase in alpha SW (i.e., ∆SW < 0) may result from alpha desynchronization triggered by eye opening, which facilitates more efficient information communication [30]. Besides, the post-eye-opening declined SW values (i.e., ∆SW > 0) in the low-frequency bands may reflect the suppression of DMN activity induced by external visual input [62]. The beta and gamma oscillations in higher frequencies are closely linked to fronto-parietal integration and local neuronal synchronization, respectively [63, 64]. The heightened sensory-driven complexity and desynchronization accompanying eye opening likely reduce local connectivity and elevate network randomness, resulting in lower SW in these bands. Notably, this frequency-dependent network reactivity was more pronounced in the right hemisphere—particularly in the delta, theta, beta1, and gamma bands—further reinforcing the right-hemispheric dominance in functional network organization.

### State-specific age effects on hemispheric EEG networks

Both hemispheres exhibited prominent SW properties in EC and EO states, with age-related effects on hemispheric small-worldness evident only in the EC state. This pattern suggests that intrinsic network organization during eyes-closed rest is more vulnerable to aging, likely because this state predominantly engages internally driven activity, such as the DMN, which has been consistently shown to decline with advancing age [65, 66]. In contrast, the EO condition is strongly influenced by continuous sensory input and externally oriented attentional processes [67], which may stabilize cortical dynamics and thereby mask subtle age-related differences [68]. The observation that ΔSW also showed significant age effects highlights that aging not only compromises baseline network efficiency but also reduces the flexibility to transition between internal and external modes of processing. Collectively, these findings indicate that intrinsic and reactive neural properties serve as more sensitive markers of aging than externally stabilized states.

Specifically, in the EC state, age-related modulation was observed in bilateral hemispheres within the theta, alpha2, and beta2 bands. SW in the 60–70 years cohort was higher in the alpha2 band than in all younger groups (20–59 years), but lower in the theta and beta2 bands relative to the 30–39 years group. According to previous literature, lower values of SW in theta and beta bands represent a sort of functional disconnection, whereas higher alpha SW may indicate inefficient cortical network performance [19, 59, 69]. Our results replicate the frequency-specific SW alterations associated with physiological aging, indicative of less efficient network topology [8, 11, 19]. Furthermore, the inclusion of middle-aged participants in this study expanded upon previous findings, indicating that such age-related effects emerge predominantly in late adulthood rather than accumulating gradually across the adult lifespan.

Regarding ΔSW, the broad age-modulatory effects across the examined frequency bands suggest that age-related influences become most pronounced during the transition from EC to EO brain state, i.e., in indices of network reactivity. In line with previous reports [8, 27, 70], the 60–70 years cohort exhibited significantly reduced network responsiveness to increased arousal relative to younger groups, with the attenuation being most prominent in the theta, alpha2, and beta2 frequency bands. Additionally, compared with the 30–39 age group, participants aged 50–59 exhibited reduced network reactivity upon eye opening (lower ΔSW) in the beta2 band and poorer cortical performance (higher SW) in the alpha2 band. Taken together, this state-dependent variability underscores the potential utility of multi-state assessments for capturing different facets of age-related SW changes. Moreover, these findings provide insights into the dynamic reorganization of aging neural networks in response to sensory input, offering implications for designing sensitive neurocognitive assessments and targeted interventions aimed at mitigating age-related functional decline.

### Age-dependent trajectories of DMN SW by frequency band

Having established hemispheric patterns of age-related alterations, we next turned to a more refined level of analysis, focusing on six hemispheric subnetworks—the left and right DMN, FN, and AN—to delineate network-specific aging effects. Despite bilateral age effects observed at the hemispheric level, significant age-dependent alterations at the subnetwork level emerged exclusively within the three right-hemispheric subnetworks. This distribution pattern of age-related effects is at least partially consistent with the right hemi-aging model, which posits greater vulnerability of right-hemispheric functions to aging [71, 72]. One plausible account of the hemispheric–subnetwork dissociation in age effects is that hemispheric SW topology captures the integrated organization of both intra- and inter-subnetwork connectivity: age-related alterations in cross-subnetwork integration or in left-hemispheric regions outside DMN/FN/AN may drive the hemispheric-level effects. The absence of subnetwork-level age effects in the left hemisphere may reflect compensatory strengthening of specific left-hemispheric pathways, given that intra-subnetwork reorganization is mainly concentrated in the right hemisphere. Future investigations leveraging decomposition of SW network into intra- versus inter-network components, high-resolution node-level mapping, and multimodal analyses integrating structural and metabolic measures will be critical to adjudicate between these alternative mechanisms. For example, it has been suggested that hemispheric differences in age-related effects are due to a smaller gray-to-white matter ratio in the right than left hemisphere [73].

Among the three subnetworks under consideration, DMN, an intrinsic FC system prominently engaged during resting states, is posited to subserve processes of mental reorganization and anticipatory readiness for goal-directed behavior [11]. It is further implicated in autobiographical memory and habitual, self-referential thought [74]. Given the predominance of alpha activity in DMN functioning [75–77], the significant age-related changes in its SW network properties—occurring exclusively in the alpha band—emerge as a predictable phenomenon. Interestingly, in the EC state, age effects on the small-worldness of the right DMN displayed completely opposite trends in low- (alpha1) and high-frequency alpha (alpha2) bands: compared to the 30–39 years group, elderly individuals aged 60–70 exhibit lower SW in the alpha1 and higher SW in the alpha2 band. There is general consensus that alpha1 and alpha2 sub-bands have different physiological meanings [78]. Alpha1 rhythm is known to be related to behavioral inhibition, and SW topological measures (namely increased C and decreased L) of DMN in the alpha1 band appear to represent significant inhibitory function [79]. Accordingly, our finding that older adults show reduced alpha1 SW in DMN may help explain the poorer inhibitory control reported in prior aging studies [80]. On the other hand, alpha2 rhythm is linked to memory and cognitive behavior [81], with higher SW in this band representing a functional counterpart of hippocampal atrophy and related-network disconnection [19, 82]. The elevated SW in the alpha2 band observed in older adults could thus be indicative of inefficient cortical performance during aging. Klimesch has emphasized that using narrower frequency bands in the study can reduce the risk that the frequency effects are canceled out or not discovered [83], which is well demonstrated in the results of alpha1 and alpha2 rhythms in this study. GAM analysis revealed that the 30–39-year cohort reached the overall peak and trough of SW across adulthood in the alpha1 and alpha2 bands, respectively, indicating optimal cortical efficiency during this age window.

For ΔSW following eye opening, the 20–29 years cohort exhibited lower reactivity in the delta band compared with the 50–70 group, and in the alpha2 band relative to the 30–39 group. In contrast, the 30–39 cohort displayed greater reactivity in the alpha2 band compared with all other age groups. The observedΔSW pattern reflects a nonlinear, age-dependent modulation of network reactivity, as further confirmed by the subsequent GAM analysis. Young adults (20–29 years) exhibited relatively low reactivity, likely reflecting the continued maturation of dynamic network regulation. The peak reactivity observed in the 30–39-year cohort, corresponding to late early adulthood, suggests a period of heightened network flexibility that is most evident in the alpha2 band associated with cognitive control and attentional engagement. This aligns with prior reports that executive functions such as working memory and inhibitory control reach their apex in the thirties before declining with age [84]. Notably, older adults (60–70 years) demonstrated markedly reduced reactivity in the alpha2 band, with ΔSW polarity fully reversed compared with younger cohorts (ΔSW > 0 versus ΔSW < 0 in other groups), reflecting impaired network adaptability and compromised dynamic reconfiguration in response to sensory input. Overall, these findings delineate distinct age- and frequency-dependent trajectories of network responsiveness across the adult lifespan.

### Age-dependent trajectories of FN SW by frequency band

FN (Broca’s area and its relationship) was selected for covering the language domain of human cognitive abilities [11]. Specifically, functional coupling between frontal regions has been shown to correlate with performance in a grammar-learning task in healthy elderly individuals [85], as well as with task-related activation during a semantic fluency paradigm and intrinsic connectivity at rest [86]. Beyond the alpha2 band, which is widely affected in hemispheric functional networks during aging, the age-related differences in the SW properties of the right FN were restricted to the beta2 and gamma frequency bands. Such a circumscribed age-related effect on a few bands in FN may be attributable to the prominent involvement of beta-gamma activities in the frontal lobe during language-related tasks [87, 88]. Considering that reduced SW properties in the beta/gamma band were linked to physiological and pathological aging [11], it can be argued that the high-frequency networks reorganization observed in the right FN may be the neural basis for the decline in language function in older adults.

For ΔSW following eye opening, ANOVA indicated greater reactivity in the alpha2 band for the 30–39 group relative to older groups (50–70 years), whereas GAM revealed a continuous, linear decline of ΔSW across adulthood (20–70 years). This discrepancy reflects methodological differences: ANOVA captures discrete group means and local fluctuations, while GAM models continuous age effects and emphasizes the overall trajectory. Collectively, these results suggest that network reactivity remains relatively high in early adulthood and declines progressively, with the 30–39 cohort representing part of this downward trajectory rather than a distinct peak. In the gamma band, the 60–70 group showed markedly reduced reactivity compared with all younger cohorts, reflecting age-related decrements in high-frequency network adaptability. Unlike the DMN, whose SW properties follow nonlinear age trajectories with peaks in late early adulthood, the FN generally shows a linear decline in small-worldness across adulthood, without a discernible developmental peak around 30–39 years.This divergence indicates that distinct cognitive networks exhibit unique patterns of adaptation and reorganization during aging, likely reflecting their functional roles and varying susceptibility to age-related decline [89].

### Age-dependent trajectories of AN SW by frequency band

AN primarily governs the cognitive properties associated with executive functions such as working memory, language, decision-making, visuospatial abilities, and also alerting, orienting, and executive control [90, 91]. Within the right AN, the age-related SW reduction in the alpha1 and gamma bands among the 60–70 group appears consistent with previous findings of declines in cognitive performance in these domains [92]. Moreover, the theta decrease and alpha2 increase in SW observed in the elderly resonate, for example, with prior observations that theta and alpha activities are often anti-correlated [93]. The alpha and theta oscillations, which have close frequency bands, could have opposite roles in working memory [94]: functional connectivity between brain regions displays increased alpha coherence with suppressed neural activity and increased theta coherence with active processing [95]. In the alpha2 band, adults aged 30–39 exhibited more efficient SW organization compared with both 20–29 and 60–70 cohorts, and demonstrated the highest ΔSW reactivity across all age groups. Mirroring the pattern seen in the DMN, this finding in the AN further supports the presence of a developmental peak in neural network efficiency during late early adulthood. This finding aligns well with previous research on the lifespan trajectory of EEG network complexity [96], which demonstrated that Shannon’s spectral entropy—an established index of EEG network complexity—peaks around ages 30–40 across frontal, parieto-occipital, and temporal regions in both hemispheres, followed by marked declines emerging in later adulthood (after approximately 50–60 years). While informative, their analysis focused exclusively on age-related changes at the regional level; our findings extend this concept to the subnetwork level, highlighting the importance of examining network-specific trajectories to better understand age-related changes in neural organization.

Another interesting finding is that in the right AN, adults aged 50–59 exhibited a transient rebound in theta and gamma SW, as well as in ΔSW within the theta band, significantly exceeding values observed in the 60–70 cohort. This pattern may reflect a temporary enhancement of neural network efficiency during midlife or an age-related, region-specific compensatory mechanism supporting the preservation of cognitive function. Further research is warranted to explore how compensatory mechanisms in specific subnetworks counteract the onslaught of aging, which may articulate ways to improve the specific functioning of older adults.

### The effects on SW cannot be simply ascribed to variations in C or L

One intriguing aspect of our findings pertains to C and the L, both of which are foundational to the computation of SW. In each recording condition, C and L did not consistently show the anticipated hemispheric differences in frequency bands with significant SW differences. These findings point to the critical point: while SW inherently relies on both C and L, the variations detected in SW do not merely recapitulate changes in these constituent metrics, but rather capture distinct aspects of functional network organization [97]. This unique sensitivity positions SW as a particularly valuable indicator for detecting subtle alterations in brain connectivity associated with aging and age-related diseases such as Alzheimer’s disease. Our results thus highlight the importance of regarding SW not simply as a derivative metric, but as a standalone measure with unique interpretive value.

### Limitations

Evidences from the present study confirm the utility to adopt a graph theory approach to investigate age-related effects on complex brain networks throughout the adult lifespan. Although the observed age-related alterations in the SW parameter yielded interesting insights, several limitations should be acknowledged given the study’s aim to provide a reliable reference dataset for clinical research and practice.

First, the age-related SW architecture changes were detected based on the cross-sectional data and thus could be potentially influenced by unbalanced cohort distributions. Because different age cohorts may be different in substantive ways, investigations of the longitudinal network dynamics should be undertaken in the future to reveal the nature of age-related changes. Second, it should be emphasized that graph metrics C and L, derived from weighted brain networks, could not be normalized against surrogate networks due to the inherent properties of the weighted connectivity values [20]. As a methodological remark, graph analysis of weighted complete graphs avoids the use of arbitrary thresholds and thus can comprehensively retain connection information. In addition, the above-mentioned limitation was alleviated by using the average values of each parameter in all frequency bands as a normalization base [11, 18–20]. Third, our investigation focused solely on SW topological measures because this study builds upon previous findings demonstrating hemispheric asymmetry in the SW properties of specific EEG networks in healthy older adults. An intriguing future study direction will be to explore whether other graph metrics have similar hemispheric asymmetry in EEG subnetworks of interest. The comparative analysis of graph measures to identify those most sensitive to age-related hemispheric changes would also be valuable. Fourth, the lack of objective cognitive data limited our ability to establish direct associations between age-related SW differences and cognitive outcomes. Future research that incorporates standardized cognitive assessment scales will be essential for elucidating the network reorganization underlying cognitive decline during physiological aging.

## Conclusion

In conclusion, graph analysis tools described here represent an interesting probe to study the distinctive features of physiological aging, with a particular focus on hemispheric functional networks that support behavior and cognition. Age-related effects were fully driven by right-hemispheric subnetworks, exhibiting modulation patterns that were both eyes-state- and frequency-specific. These findings offer novel insights into the developmental and aging trajectories of functional networks from two complementary perspectives—the intrinsic efficiency of hemispheric networks and their adaptability in shifting between internally and externally oriented modes of processing. Furthermore, the electrode configuration used in this study closely aligns with practical clinical settings, enabling the establishment of an eyes-state-dependent baseline that may facilitate more precise and context-aware interpretation of hemispheric network topology in clinical practice.

## Data Availability

The dataset analyzed in this study is publicly available on OpenNeuro: Resting-state EEG data before and after cognitive activity across the adult lifespan and a 5-year follow-up (OpenNeuro dataset ds005385, version 1.0.2)  https://doi.org/10.18112/openneuro.ds005385.v1.0.2.
